# Orthostatic Autonomic Dysregulation and Orthostatic Intolerance Following Adult Concussion

**DOI:** 10.1001/jamanetworkopen.2026.41160

**Published:** 2026-10-22

**Authors:** Veronik Sicard, Courtney Kennedy, Lauren Xinyue Duan, Andrée-Anne Ledoux, John J. Leddy, Kim Connelly, Achelle Cortel-LeBlanc, Stephen A. Kutcher, Lawrence Richer, Richard J. Webster, Charlotte Anderson, Sharon Johnston, Roger Leonard Zemek

**Affiliations:** 1 Faculty of Nursing, Université Laval, Québec, Québec, Canada; 2 Faculty of Kinesiology, Sport, and Recreation, University of Alberta, Edmonton, Alberta, Canada; 3 Children’s Hospital of Eastern Ontario Research Institute, Ottawa, Ontario, Canada; 4 University of Ottawa Brain and Mind Research Institute, University of Ottawa, Ottawa, Ontario, Canada; 5 Department of Neuroscience, Carleton University, Ottawa, Ontario, Canada; 6 Department of Cellular and Molecular Medicine, University of Ottawa, Ottawa, Ontario, Canada; 7 UBMD Department of Orthopaedics and Sports Medicine, Jacobs School of Medicine and Biomedical Sciences, State University of New York at Buffalo, Buffalo; 8 Keenan Research Center for Biomedical Science, Li Ka Shing Knowledge Institute, St Michael’s Hospital, Unity Health Toronto, University of Toronto, Toronto, Ontario, Canada; 9 Division of Cardiology, St Michael’s Hospital, Unity Health Toronto, University of Toronto, Toronto, Ontario, Canada; 10 Institut du Savoir Montfort, Ottawa, Ontario, Canada; 11 Faculty of Medicine, University of Ottawa, Ottawa, Ontario, Canada; 12 Women and Children’s Health Research Institute and Faculty of Medicine and Dentistry, College of Health Sciences, University of Alberta, Edmonton, Alberta, Canada; 13 Telfer School of Medicine, University of Ottawa, Ottawa, Ontario, Canada; 14 Alpha Health Services, Toronto, Ontario, Canada; 15 Department of Physiotherapy, University of Toronto, Ontario, Canada.; 16 Department of Family Medicine, University of Ottawa, Ottawa, Ontario, Canada; 17 Department of Pediatrics, University of Ottawa, Ottawa, Ontario, Canada

## Abstract

This cohort study examines the prevalence and co-occurrence of autonomic dysregulation and orthostatic intolerance among adults with concussion and explores associated demographic and injury-related factors.

## Introduction

Concussion can impair autonomic nervous system regulation, presenting as autonomic dysregulation (AD), which is excessive physiological reactivity to postural change, or orthostatic intolerance (OI), which is symptom exacerbation on standing (eg, headache, dizziness, or nausea). As autonomic symptoms overlap with core concussion symptoms, AD and OI are plausible contributors to persisting symptoms after concussion and recovery variability. Few studies examined orthostatic AD after concussion, most in pediatric cohorts, and none used the criterion standard head-up tilt table test (HUTT) in adults.^1,2,3,4,5^ Current concussion guidelines recommend AD and OI assessment as part of a comprehensive framework,^6^ yet AD and OI in adult concussion remain poorly characterized. Our objectives were to estimate the prevalence and co-occurrence of AD and OI during a 2-minute HUTT in adults with concussion, and to explore their associations with key demographic and injury-related characteristics.

## Methods

This prospective cohort TRANSCENDENT^7^ substudy was conducted within an Ontario network of physician-led interdisciplinary concussion clinics operating as a learning health system. We included patients aged 18 to 85 years presenting to the clinics within 3 months of their diagnosed concussion (June 12, 2022, to October 30, 2025). All participants provided written informed consent. See the eMethods in Supplement 1 for details. The study was approved by the Hôpital Montfort Research Ethics Board (22-23-09-028) and CHEO Research Ethics Board (No. 24/17X) and is reported per the STROBE reporting guidelines.

Demographics, medical and injury history, and current symptom burden (Post-Concussion Symptom Scale; 22 items rated 0-6; total score, 0-132, with higher scores denoting greater symptom burden)^6^ were prospectively collected via standardized forms within the Collaborative Health Record (Telus). Consenting participants underwent HUTT using an electric tilt table; after 2-minute supine rest, systolic blood pressure (SBP), diastolic blood pressure (DBP), and heart rate (HR) were recorded. Participants were then tilted to 70° (for approximately 15 seconds) and held upright, with SBP, DBP, HR, and orthostatic symptoms (headache, dizziness, light-headedness, blurry vision, weakness, pre-syncope, and confusion) recorded at 15 seconds, 1 minute, and 2 minutes.

AD was defined as meeting at least 1 consensus criterion at 15 seconds, 1 minute, or 2 minutes (relative to baseline): (1) SBP decrease at least 20 mm Hg, (2) DBP decrease at least 10 mm Hg, and (3) HR increase at least 40 beats/minute (ages 16-19 years) or at least 30 beats/minute (age >19 years).^8,9^ OI was defined as new or worsening symptoms at any time point upon tilting.

Wilson scores with 95% CIs were used to estimate physiological AD, OI, and their co-occurrence prevalence. Univariate models explored associations between demographics and injury characteristics and AD and/or OI. Analyses used R statistical software version 4.3.1 (R Project for Statistical Computing). Two-sided *P* < .05 was significant.

## Results

Of 597 participants (median [IQR] age, 34.00 [26.00-46.00] years; 397 [66.8%] female; median [IQR] time from injury, 20.00 [11.00-38.00] days), physiological AD occurred in 107 of 597 patients (17.92%; 95% CI, 15.06%-21.20%) and OI in 347 of 597 patients (58.12%; 95% CI, 54.13%-62.02%). Among the 387 participants with either physiological AD and or OI, co-occurrence was limited to 67 of 387 patients (17.31%; 95% CI, 13.87%-21.40%). AD only occurred in 40 of 387 patients (10.34%; 95% CI, 7.68%-13.77%), and OI only occurred in 280 of 387 patients (72.35%; 95% CI, 67.69%-76.57%).

Participants with physiological AD were younger than those without AD (median [IQR] age, 28.00 [23.00-44.00] vs 35.00 [26.00-46.00] years; *P* = .01) (Table). Participants with OI were more likely than those without OI to be female (249 of 347 patients [72.17%] vs 148 of 250 patients [59.44%]; *P* = .001), to have a preinjury diagnosis of mental health disorder (150 of 347 patients [43.23%] vs 88 of 250 patients [35.20%]; *P* = .048) and migraines (61 of 347 patients [17.58%] vs 29 of 250 patients [11.60%]; *P* = .04), and to report greater symptom burden (median [IQR] score, 54.00 [32.00-74.00] vs 66.00 [48.00-86.00]; *P* < .001). Participants with OI were more likely than those without OI to report confusion (192 of 347 patients [56.80%] vs 102 of 250 patients [41.80%]; *P* = .001) and answering questions slowly at the time of injury (159 of 347 patients [46.90%] vs 97 of 250 patients [39.59%]; *P* = .04) (Table).

**Table.** Association of Physiological Autonomic Dysregulation and Orthostatic Intolerance With Key Demographic and Injury Variables

Characteristics	Physiological autonomic dysfunction			Orthostatic intolerance		
	Patients, No. (%)		*P* value	Patients, No. (%)		*P* value
	Present (n = 107)	Absent (n = 490)		Present (n = 347)	Absent (n = 250)	
Age, median (IQR), y	28.00 (23.00-44.00)	35.00 (26.00-46.00)	.01^a^	33.00 (25.00-44.00)	36.00 (26.00-49.00)	.08
Sex						
Female	69 (64.49)	328 (67.35)	.57	249 (71.17)	148 (59.44)	.001^a^
Male	38 (35.51)	159 (32.65)		96 (21.83)	101 (40.56)	
Missing, No.	0	3		2	1	
Neurodevelopment disorder^b^	21 (19.63)	80 (16.33)	.41	62 (17.87)	39 (15.60)	.47
Mental health disorder^c^	38 (35.51)	200 (40.82)	.31	150 (43.23)	88 (35.20)	.048^a^
Migraines	11 (10.28)	79 (16.12)	.13	61 (17.58)	29 (11.60)	.04^a^
Previous concussion	65 (60.75)	263 (53.67)	.18	194 (55.91)	134 (53.60)	.58
Mechanism						
Assault	5 (4.67)	10 (2.04)	.52	12 (3.46)	3 (1.20)	.18
Motor vehicle collision	19 (17.76)	107 (21.84)		79 (22.77)	47 (18.80)	
Nonsports	39 (36.45)	174 (35.51)		125 (36.02)	88 (35.20)	
Sports	24 (22.43)	105 (21.43)		67 (19.31)	62 (24.80)	
Unknown or unclear	20 (18.69)	94 (19.18)		64 (18.44)	50 (20.00)	
Loss of consciousness						
Yes	13 (12.50)	64 (13.47)	.96	45 (13.43)	32 (13.11)	.13
No	76 (73.08)	345 (72.63)		253 (70.15)	186 (76.23)	
Unsure	15 (14.42)	66 (13.89)		55 (16.42)	26 (10.66)	
Missing, No.	3	15		12	6	
Posttraumatic amnesia						
Yes	28 (26.92)	126 (26.53)	.89	94 (28.06)	60 (24.59)	.22
No	58 (55.77)	257 (54.11)		172 (51.34)	143 (58.61)	
Unsure	18 (17.31)	92 (19.37)		69 (20.60)	41 (16.80)	
Missing, No.	3	15		12	16	
Confused						
Yes	54 (53.47)	240 (49.90)	.43	192 (56.80)	102 (41.80)	.001^a^
No	39 (38.61)	181 (37.63)		108 (31.95)	112 (45.90)	
Unsure	8 (7.92)	60 (12.47)		38 (11.24)	30 (12.30)	
Missing, No.	6	9		9	6	
Answered questions slowly						
Yes	47 (44.34)	209 (43.72)	.99	159 (46.90)	97 (39.59)	.04^a^
No	37 (34.91)	169 (35.36)		105 (30.97)	101 (41.22)	
Unsure	22 (20.75)	100 (20.92)		75 (22.12)	47 (19.18)	
Missing, No.	1	12		8	5	
Time since injury, median (IQR), d	19.00 (9.00-38.00)	21. 00 (12.00-38.00)	.34	21.00 (12.00-39.00)	19.00 (11.00-38.00)	.30
Symptom score, median (IQR)	59.50 (40.00-85.00)	62.00 (40.00-81.00)	.79	66.00 (48.00-86.00)	54.00 (32.00-74.00)	<.001^a^
Missing, No.	9	52	NA	48	48	NA

Abbreviation: NA, not applicable.

^a^ Denotes statistical significance at *P* < .05.

^b^ Includes a previous diagnosis of attention-deficit disorder with or without hyperactivity, learning disabilities, and other developmental disorders.

^c^ Includes a previous diagnosis of anxiety, depression, sleep disorder, or other mental health disorder.

## Discussion

In this cohort study, physiological AD (1 in 5 participants) and OI (3 in 5) co-occurred in only about one-fifth of cases, suggesting that these are related but dissociable processes following concussion. This is consistent with prior pediatric work,^1^ despite differences in population and protocol, and suggests that physiological AD is neither necessary nor sufficient for OI.

Clinical profiles associated with physiological AD and OI differed: AD was associated only with younger age, whereas OI was associated with female sex, preinjury mental health and migraine diagnoses, greater current symptom burden, and markers of acute injury. This divergence suggests that OI is more closely tied to injury characteristics and subjective symptom expression than to measurable autonomic change, consistent with prior evidence linking symptom provocation during a stress test to anxiety, catastrophizing, and somatization rather than physiopathology alone.^10^

Strengths of this study include enrollment without preselection for suspected autonomic challenges, large sample size, and use of a standardized, comprehensive AD assessment protocol. Limitations include the absence of a control group or preinjury measurements. The relatively young cohort age limits generalizability to older adults. We were unable to systematically verify medication data from a pragmatic clinical data source. Requiring complete orthostatic measurements may have introduced selection bias. Limited numbers meeting AD and/or OI criteria precluded multivariable modeling; univariate findings should be interpreted as hypothesis-generating. The study nonetheless provides prevalence estimates to inform the design and powering of future studies.

Our findings argue against using OI as a surrogate for physiological AD, or vice versa: relying on either domain alone risks underrecognizing clinically relevant dysregulation. Clinically, AD, psychological factors, and physiological deconditioning may represent interrelated and modifiable targets for supported, progressive exercise, although careful titration remains essential given this population’s symptom sensitivity.

## Notes

Supplement 1. eMethods. eReferences

Supplement 2. Nonauthor Collaborators. uOttawa Brain-Heart Interconnectome (BHI) and TRANSCENDENT Concussion Integrated Discovery Programs Members

Supplement 3. Data Sharing Statement
