# Supplementary material for: Orthostatic Autonomic Dysregulation and Orthostatic Intolerance Following Adult Concussion
Source: JAMA Netw Open. 2026 Oct 22;9(10):e2641160. doi: 10.1001/jamanetworkopen.2026.41160 (PMC13691653; doi:10.1001/jamanetworkopen.2026.41160)
Supplement: Supplementary file 1 [file jamanetwopen-e2641160-s001.pdf]

Supplemental Online Content

Sicard V, Kennedy C, Duan LX, et al; uOttawa Brain-Heart Interconnectome (BHI) and TRANSCENDENT Concussion Integrated Discovery Programs. Orthostatic autonomic dysregulation and orthostatic intolerance following adult concussion. *JAMA Netw Open*. 2026;9(10):e2641160. doi:10.1001/jamanetworkopen.2026.41160

eMethods.
eReferences

This supplemental material has been provided by the authors to give readers additional information about their work.

© 2026 Sicard V et al. *JAMA Network Open*.

eMethods

Study Design

This prospective cohort study is conducted in two sequential phases within a network of physician-led concussion clinics (360 Concussion Care) operating as a learning health system. Both phases (embedded in the TRANSCENDENT research program[1]) used harmonized protocols and recruited patients with acute to persistent concussions of all mechanisms of injury. Phase 1 enrolled participants at the Ottawa, ON, site from June 12, 2022, to May 4, 2024. With additional funding, the Brain Health Interconnectome (BHI)-TRANSCENDENT[1] added a site (Mississauga, ON) and expanded data collection (e.g., additional biomarkers; outside the scope of this manuscript) for Phase 2. Phase 2 launched July 18, 2024, in Ottawa and January 7, 2025, in Mississauga; participants from inception to October 30, 2025, are included in this study. The testing protocol was standardized between sites.

Phase 1 was approved by the Hôpital Montfort Research Ethics Board (22-23-09-028), and Phase 2 by the Children's Hospital of Eastern Ontario Research Ethics Board (#24/17X). The study was conducted in accordance with the Declaration of Helsinki and according to Canadian and international standards of Good Clinical Practice, and is reported per the STROBE guidelines.[2]

Participants

In both phases, patients presenting to the concussion clinics were approached for research participation. In Phase 1, patients presenting to the Ottawa clinic were pre-screened using their medical chart information filled out before the scheduled appointment, then screened in person by the study team and completed a shortened research protocol if they consented. In Phase 2 (TRANSCENDENT[1]), all patients presenting to the clinics were approached for participation in the broader TRANSCENDENT research program through pre-visit questionnaires integrated into the clinic's electronic medical record system, with options for e-consent or in-person discussion with the research team. Participants could subsequently participate in specific substudies if they met the respective substudy eligibility criteria. For the orthostatic testing substudy, exclusion criteria were: severe chronic neurological developmental delay resulting in communication difficulties; clinically significant neurological disease that may affect the ANS (i.e., structural cerebral abnormalities, cerebral palsy, congenital central hypoventilation, Prader-Willi syndrome, Riley-Day syndrome, spinal cord lesion, other peripheral neuropathy, or moderate-to-severe

dementia); history of unstable cardiovascular disease (e.g., stroke, transient ischemic attacks, myocardial infarction, unstable angina, heart failure, atrial fibrillation, or other cardiac arrhythmias) in the past three months requiring hospital admission, unable to tolerate lying or standing on tilt table (e.g., lower-limb orthopedic injury); unable to provide proper written informed consent.

For the present analysis, participants were included if they were aged 18-85 years, had a physician-diagnosed concussion (confirmed by the clinic's physicians), presented to the clinic within 90 days of injury, and completed the orthostatic testing protocol. No minimum symptom burden was required, and participants were not required to have persisting symptoms after concussion.

Procedures

Patient demographic information, medical and injury history, and current symptom burden on the Post-Concussion Symptom Scale (PCSS; 22 items rated 0-6, total score 0-132) from the Sport Concussion Assessment Tool (SCAT-5/6)[3,4] were primarily collected through self-reported questionnaires completed via the clinic's electronic medical records system before their visit. When required, these data were completed or verified by physicians or trained research assistants (RAs) during the clinical encounter.

Orthostatic Testing Protocol

RAs subsequently conducted orthostatic testing. Participants underwent 70° HUTT using an electric tilt table. Participants were instructed to remain as still and silent as possible, breathing normally with calm, deep breaths throughout the protocol. Following a 2-minute supine rest, baseline measurements were recorded (i.e., BP and HR via medical-grade automatic BP monitors (Welch Allyn ProBP line, New York, United States). Participants were then tilted to 70° over approximately 15 seconds and maintained in the upright position. Serial measurements were recorded immediately upon upright positioning (approximately 15 seconds), then at 1-minute intervals thereafter for either 2 minutes (Phase 1) or 3 minutes (Phase 2), after which participants were returned to supine. The additional 3-minute assessment in Phase 2 was introduced based on Phase 1 observations that the cumulative proportion of participants meeting AD criteria continued to increase through 2 minutes. To ensure comparability across phases, the present analyses were restricted to the 15-second, 1-minute, and 2-minute timepoints available in both phases; the Phase 2 protocol was standardized across participating sites. Orthostatic symptoms, including headache,

© 2026 Sicard V et al. *JAMA Network Open*.

dizziness, light-headedness, blurry vision, nausea, weakness, pre-syncope, and confusion, were recorded at the same timepoints. Following completion of orthostatic testing, participants were returned to the supine position.

Outcome Measures

AD was operationally defined as meeting any one of the following consensus-defined clinical criteria upon tilting (15-sec, 1-minute, and 2-minute), relative to rest: 1) ≥20 mmHg decrease in SBP,[5,6] 2) ≥10 mmHg decrease in DBP,[5,6] or 3) ≥40 bpm increase in HR for ages 16-19[5,7,8] and ≥30 bpm for 19 and older.[5,7,8]. These criteria characterize orthostatic HR and BP responses and do not represent direct measurement of sympathetic or parasympathetic nervous system activity.

Orthostatic intolerance (OI) was defined as new or worsening symptoms occurring upon tilting. Specifically, at each timepoint (15-sec, 1-minute, and 2-minute), participants were systematically assessed for the eight orthostatic symptoms listed above. OI was operationally defined as the emergence of a new orthostatic symptom or worsening of a previously reported symptom at any assessed timepoint compared to rest.

Statistical Analyses

Descriptive statistics summarized patient demographics, medical histories, injury characteristics, and AD and symptom provocation characteristics. Wilson scores with 95% CIs were used to describe the overall prevalence of AD and OI. Descriptive analysis also examined the overlap between AD and OI. Univariate models (Wilcoxon rank sum test, Pearson's Chi-squared test, or Fisher's exact test, as appropriate) explored associations of demographics, injury characteristics, and both AD and OI. No corrections for multiple comparisons were applied due to the exploratory nature of the analysis. Missing data primarily affected demographic and medical and injury history obtained from the pragmatic clinical data source. Analyses were conducted using available cases; missing data were not imputed. Analyses were performed using R statistical software v4.3.1,[9] and p-values<0.050 were considered significant.

No a priori sample size calculation was performed for the present analysis because reliable estimates of the expected prevalence of physiological AD and OI in adults with concussion undergoing HUTT were unavailable. All eligible participants who completed HUTT during the prespecified study period were included to maximize the precision of the prevalence estimates.

© 2026 Sicard V et al. *JAMA Network Open*.

eReferences

1. Zemek, R. *et al.* TRANSCENDENT (Transforming Research by Assessing Neuroinformatics across the Spectrum of Concussion by Embedding iNterdisciplinary Data-collection to Enable Novel Treatments): protocol for a prospective observational cohort study of concussion patients with embedded comparative effectiveness research within a network of learning health system concussion clinics in Canada. *BMJ Open* **15**, e095292 (2025).
2. von Elm, E. *et al.* The Strengthening the Reporting of Observational Studies in Epidemiology (STROBE) statement: guidelines for reporting observational studies. *Lancet Lond. Engl.* **370**, 1453–1457 (2007).
3. Patricios, J. S. *et al.* Consensus statement on concussion in sport: the 6th International Conference on Concussion in Sport-Amsterdam, October 2022. *Br. J. Sports Med.* **57**, 695–711 (2023).
4. Medicine, B. P. G. L. and B. A. of S. and E. Sport concussion assessment tool - 5th edition. *Br. J. Sports Med.* **51**, 851–858 (2017).
5. Freeman, R. *et al.* Consensus statement on the definition of orthostatic hypotension, neurally mediated syncope and the postural tachycardia syndrome. *Clin. Auton. Res. Off. J. Clin. Auton. Res. Soc.* **21**, 69–72 (2011).
6. The Consensus Committee of the American Autonomic Society and the American Academy of Neurology. Consensus statement on the definition of orthostatic hypotension, pure autonomic failure, and multiple system atrophy. The Consensus Committee of the American Autonomic Society and the American Academy of Neurology. *Neurology* **46**, 1470 (1996).

© 2026 Sicard V et al. *JAMA Network Open*.

7. Sheldon, R. S. *et al.* 2015 heart rhythm society expert consensus statement on the diagnosis and treatment of postural tachycardia syndrome, inappropriate sinus tachycardia, and vasovagal syncope. *Heart Rhythm* **12**, e41-63 (2015).
8. Singer, W. *et al.* Postural Tachycardia in Children and Adolescents – What is Abnormal? *J. Pediatr.* **160**, 222–226 (2012).
9. R Core Team. R: A language and environment for statistical computing. R Foundation for Statistical Computing (2022).

© 2026 Sicard V et al. *JAMA Network Open*.
