# Supplementary material for: Orthostatic Autonomic Dysregulation and Orthostatic Intolerance Following Adult Concussion
Source: JAMA Netw Open. 2026 Oct 22;9(10):e2641160. doi: 10.1001/jamanetworkopen.2026.41160 (PMC13691653; doi:10.1001/jamanetworkopen.2026.41160)
Supplement: Supplementary file 2 [file jamanetwopen-e2641160-s002.pdf]

Supplemental Online Content: Nonauthor Collaborators

*Indicates required information. Only first name, last name, and suffix will appear in PubMed.

*Group Name(s): TRANSCENDENT Concussion Integrated Discovery Program and uOttawa Brain-Heart Interconnectome (BHI) Program								
***First Name and Middle Initial(s)**	***Last Name**	***Suffix (eg, Jr, III)**	Academic Degrees	Institution	Location (city, state/province, country)	Role or Contribution, eg, chair, principal investigator	Group (if more than 1 Group listed in the byline) and/or Subgroup (eg, Steering Committee)	
Noah	Silverberg		PhD	Faculty of Medicine, The University c	Vancouver, British Columb	Co-Principal Investigator		
Keith Owen	Yeates		PhD	Alberta Children's Hospital Research	Calgary, Alberta, Canada	Co-Principal Investigator		
Monica	Lamoureux		MSc	Children's Hospital of Eastern Ontari	Ottawa, Ontario, Canada	Director of Research Operations		
Lisa	Albrecht		MSc	Children's Hospital of Eastern Ontari	Ottawa, Ontario, Canada	Research Project Manager		
Andrew	Dodd		MS	Children's Hospital of Eastern Ontari	Ottawa, Ontario, Canada	Research Associate		
Ewa	Sucha		MSc	Children's Hospital of Eastern Ontari	Ottawa, Ontario, Canada	Methodologist and Biostatistician		
Ivan	Terekhov		BS	Children's Hospital of Eastern Ontari	Ottawa, Ontario, Canada	Director of Research Informatics, AI and Technology		
Jacqueline	van Ierssel		PT, PhD	Children's Hospital of Eastern Ontari	Ottawa, Ontario, Canada	Research Associate		
Andra	Smith		PhD	School of Psychology, University of C	Ottawa, Ontario, Canada	Collaborator		
Jennifer	Reed		PhD	Faculty of Medicine, University of Ottawa	Ottawa, Ontario, Canada	Collaborator		
Lisa	Walker		PhD	Faculty of Medicine, University of Ot	Ottawa, Ontario, Canada	Collaborator		
Rebekah	Mannix		MD	Division of Emergency Medicine, Bos	Boston, Massachusetts	Collaborator		
Christina	Master		MD	Division of Pediatric Orthopaedics, Tl	Philadelphia, Pennsylvania	Collaborator		
Steven	Hicks		PhD	Department of Pediatrics, Penn State	Hershey, Pennsylvania	Collaborator		
