# Supplementary material for: Orthostatic Autonomic Dysregulation and Orthostatic Intolerance Following Adult Concussion
Source: JAMA Netw Open. 2026 Oct 22;9(10):e2641160. doi: 10.1001/jamanetworkopen.2026.41160 (PMC13691653; doi:10.1001/jamanetworkopen.2026.41160)
Supplement: Supplementary file 3 [file jamanetwopen-e2641160-s003.pdf]

Data Sharing Statement

Sicard. Orthostatic Autonomic Dysregulation and Orthostatic Intolerance Following Adult Concussion. *JAMA Netw Open*. Published October 26, 2026.
doi:10.1001/jamanetworkopen.2026.41160

Data
Data available: Yes
Data types: Deidentified participant data
How to access data: rzemek@cheo.on.ca
When available: With publication

Supporting Documents
Document types: None

Additional Information
Who can access the data: Data will be made available following reasonable request to the corresponding author, and data will be shared publicly on the Ontario Brain Institutes BrainCODE data platform.
Types of analyses: Any purpose.
Mechanisms of data availability: After approval or a proposal
